# Interpretation of molecular autopsy findings in 45 sudden unexplained death cases: from coding region to untranslated region

**DOI:** 10.1007/s00414-024-03329-6

**Published:** 2024-09-13

**Authors:** Shouyu Wang, Jianghua Du, Qi Shen, Cordula Haas, Jacqueline Neubauer

**Affiliations:** 1 Department of Forensic Medicine, School of Basic Medical Sciences, Fudan University, Shanghai, China; 2 Zurich Institute of Forensic Medicine, University of Zurich, Zurich, Switzerland

**Keywords:** Sudden unexplained death, Molecular autopsy, Untranslated regions, Whole-exome sequencing, Dual-luciferase reporter assay

## Abstract

Sudden unexplained death (SUD) can affect apparently healthy adolescents and young adults with no prior clinical symptoms and no clear diagnostic findings at autopsy. Although primary cardiac arrhythmias have been shown to be the direct cause of death in the majority of SUD cases, the genetic predisposition contributing to SUD remains incompletely understood. Currently, molecular autopsy is considered to be an effective diagnostic tool in the multidisciplinary management of SUD, but the analysis focuses mainly on the coding region and the significance of many identified variants remains unclear. Recent studies have demonstrated the strong association between human disease and genetic variants in untranslated regions (UTRs), highlighting the potential role of UTR variants in the genetic predisposition to SUD. In this study, we searched for UTR variants with likely functional effects in the exome data of 45 SUD cases. Among 244 genes associated with cardiac diseases, three candidate variants with high confidence of pathogenicity were identified in the UTRs of *SCO2*, *CALM2* and *TBX3* based on a rigorous filtering strategy. A functional assay further validated the effect of these candidate variants on gene transcriptional activity. In addition, the constraint metrics, intolerance indexes, and dosage sensitivity scores of genes affected by the candidate variants were considered when estimating the consequence of aberrant gene expression. In conclusion, our study presents a practical strategy for UTR variant prioritization and functional annotation, which could improve the interpretation of molecular autopsy findings in SUD cohorts.

**Supplementary Information:**

The online version contains supplementary material available at 10.1007/s00414-024-03329-6.

## Introduction

Unexpected sudden natural death in young individuals can very often be the first reported manifestation and most severe outcome of an undiagnosed disease. For the majority of cases, the cause of death turns out to be congenital cardiovascular diseases [1, 2]. However, there is still around one third of the cases that remain elusive and are termed as sudden unexplained death (SUD). In these cases, no or only mild histological changes can be identified through a comprehensive medico-legal investigation [3, 4]. In the past, a growing number of studies have demonstrated that primary cardiac arrhythmias, such as Brugada syndrome (BrS), long QT syndrome (LQTS), and short QT syndrome (SQTS), account for the majority of SUD cases. In addition, inherited cardiomyopathies, such as dilated cardiomyopathy, hypertrophic cardiomyopathy, and restrictive cardiomyopathy, could also lead to lethal arrhythmias in the early stages when only non-specific morphological features can be observed [5–7].

Currently, effective prediction algorithms for the functional annotation of variants located in coding regions or canonical splice sites have greatly facilitated the application of molecular autopsy in the multidisciplinary management of SUD [8, 9]. Nevertheless, due to a variety of regulatory mechanisms behind the non-coding sequences and the poor understanding of dosage-sensitive genes, the interpretation of rare variants in the non-coding region of known SUD susceptibility genes remains challenging. To better elucidate the genetic background of SUD, there is a need for the development of specialized guidelines that allow appropriate prioritization and functional annotation of non-coding variants.

Since the untranslated regions (UTRs) of mRNAs contain various regulatory elements, rare variants in the 5’ UTR and 3’ UTR are usually considered an important class of non-coding variants with significant impact on post-transcriptional and translational processes [10]. In a recent study, Griesemer et al. applied a massively parallel reporter assay to systematically evaluate the functional effects of genetic variations in 3’ UTRs. Their work nominated hundreds of novel 3’ UTR causal variants with genetically fine-mapped phenotype associations, once again highlighting the strong association between UTR variants and human disease [11]. However, only a handful of SUD studies have taken UTR variants into consideration. Therefore, compared to coding variants, a much smaller number of UTR variants have been reported to modify the risk of cardiac arrhythmia or SUD [12–16].

In this study, we re-evaluated the whole-exome sequencing (WES) data in a SUD cohort of 45 individuals by investigating the functional effects of variants in the UTRs of 244 genes associated with cardiac diseases. Subsequently, updated criteria for variant screening were applied, where ACMG/AMP rules for the pathogenicity assessment and functional prediction for the recognition of regulatory elements-involved regions were both taken into consideration. The impact of variants that met our requirements on gene transcriptional activity was validated by an additional functional assay. In order to establish a direct connection between our candidate variants and the genetic predisposition to SUD, we have further estimated the consequences of aberrant gene expression based on the constraint metrics, intolerance indexes, and dosage sensitivity scores.

## Materials and methods

### WES data re-analysis

The WES data of 45 SUD cases from our previous work [17, 18] were re-evaluated for the purpose of this study. Since the Sure Select All Exon V5 + UTR kit (Agilent Technologies AG, Basel, Switzerland) was used for library preparation, identification of variants in the UTRs of target genes was available. The inclusion criteria, autopsy findings and sequencing procedures have been described previously. The SUD cohort consisted of 45 cases with a mean age (± SD) of 30.2 (± 14.5) years (range: 1–63 years of age) and a mean body mass index (± SD) of 24.9 (± 4.9) (range: 13.7–35.5). 34 (76%) of the deceased were males and most of them were of European origin (89%).

Genetic investigation of the WES data was confined to a target gene panel consisting of 244 genes associated with cardiac diseases (Supplementary Table S1). This list of candidate genes is a combination of genes reported to be associated with heart diseases in various SCD/SUD studies and is based on genes listed in the recommendations and guidelines for genetic testing in sudden cardiac death cases [19–22]. Since the pathogenicity assessment of coding region/splice site variants identified in these genes was conducted previously [18], we herein investigated the functional effects of variants in the UTRs of these genes.

Variant screening was performed according to the following rules: (1) allele frequency-based filtration retains only rare variants with a global minor allele frequency (MAF) < 0.1% (including variants with unknown MAF) according to the Genome Aggregation Database (gnomAD) [23], (2) pathogenicity assessment-based filtration retains only variants classified as being pathogenic, likely pathogenic, or variants of uncertain significance (VUS) following the ACMG/AMP guidelines [24, 25], and (3) functional prediction-based filtration retains only variants with likely regulatory effect. In this third step, we used Ensembl Variant Effect Predictor (VEP) [26], miRDB [27], SRAMP [28], and rSNPBase [29] to identify variants located on predicted regulatory elements, including upstream premature start codon (uAUG), Kozak consensus sequence, miRNA binding site, m6A site, and other functional-related regions. In addition, circVIS was used to visualize the overlapping sequences between the variants and known human circRNAs [30].

### Plasmid construction and dual-luciferase reporter assay

The full length 5’ UTR of *SCO2*, 3’ UTR of *CALM2*, and 3’ UTR of *TBX3* containing either the mutant type (MT) sequence with the candidate variants or wild type (WT) sequences were directly synthesized by GENEWIZ (Azenta Life Sciences) and subcloned into the BglII and HindIII sites (for 5’ UTR), or the XbaI and FseI sites (for 3’ UTR) of pGL3-Basic plasmid (Promega), generating the WT and MT plasmids (Supplementary Table S2).

In-vitro experiments were conducted on two different cell lines (HEK293 and AC16) in parallel to increase the validity of our results. Both cell lines were cultured in DMEM supplemented with 10% FBS at 37℃ in a cell incubator supplemented with 5% CO_2_. Transfection of the plasmids was performed in 24-well plates (Corning) using Lipofectamin 2000 (Invitrogen) according to the manufacturer’s protocol. In each well, 450 ng of the reconstructed pGL3-Basic plasmid was co-transfected with 50 ng of pRL-TK plasmid (Promega). In addition, an empty pGL3-Basic/pRL-TK co-transfected group was used as the negative control. Twenty-four hours after transfection, cells were harvested immediately with the addition of 100 µL passive lysis buffer per well. The firefly luciferase activity normalized by the renilla luciferase activity was measured with the Synergy H4 microplate reader (BioTek) using the dual-luciferase reporter assay System (Promega). All experiments were repeated three times and each plasmid group was triplicated in three wells.

### Estimating the consequence of aberrant gene expression

Pathogenic variants in the UTRs usually play a critical role in regulating the expression of genes, resulting in similar outcomes of gene dosage effect caused by loss-of-function (LOF) and gain-of-function (GOF) variants. We have therefore investigated the consequence of gene expression change observed in the dual-luciferase reporter assay results. To achieve a comprehensive assessment of the association between known LOF and GOF variants in these genes and selective pressure, several disease-related properties of the three genes affected (*SCO2*, *CALM2* and *TBX3*) were evaluated, including the constraint metrics (pLI and LOEUF) [31], intolerance indexes (ncRVIS and ncGERP) [32], and dosage sensitivity scores obtained from the ClinGen Genome Dosage Map (http://www.ncbi.nlm.nih.gov/projects/dbvar/clingen/).

### Statistical analysis

Statistical analyses were implemented with GraphPad Prism software v 8.3.0. The relative luciferase activities were presented as mean ± standard error of mean (SEM). Student’s *t* test was used to examine the differences of the relative luciferase activities between WT and MT. All statistical tests were two-sided and a *p* value < 0.05 was considered statistically significant.

## Results

### Variants with likely regulatory effects

**Fig. 1:**
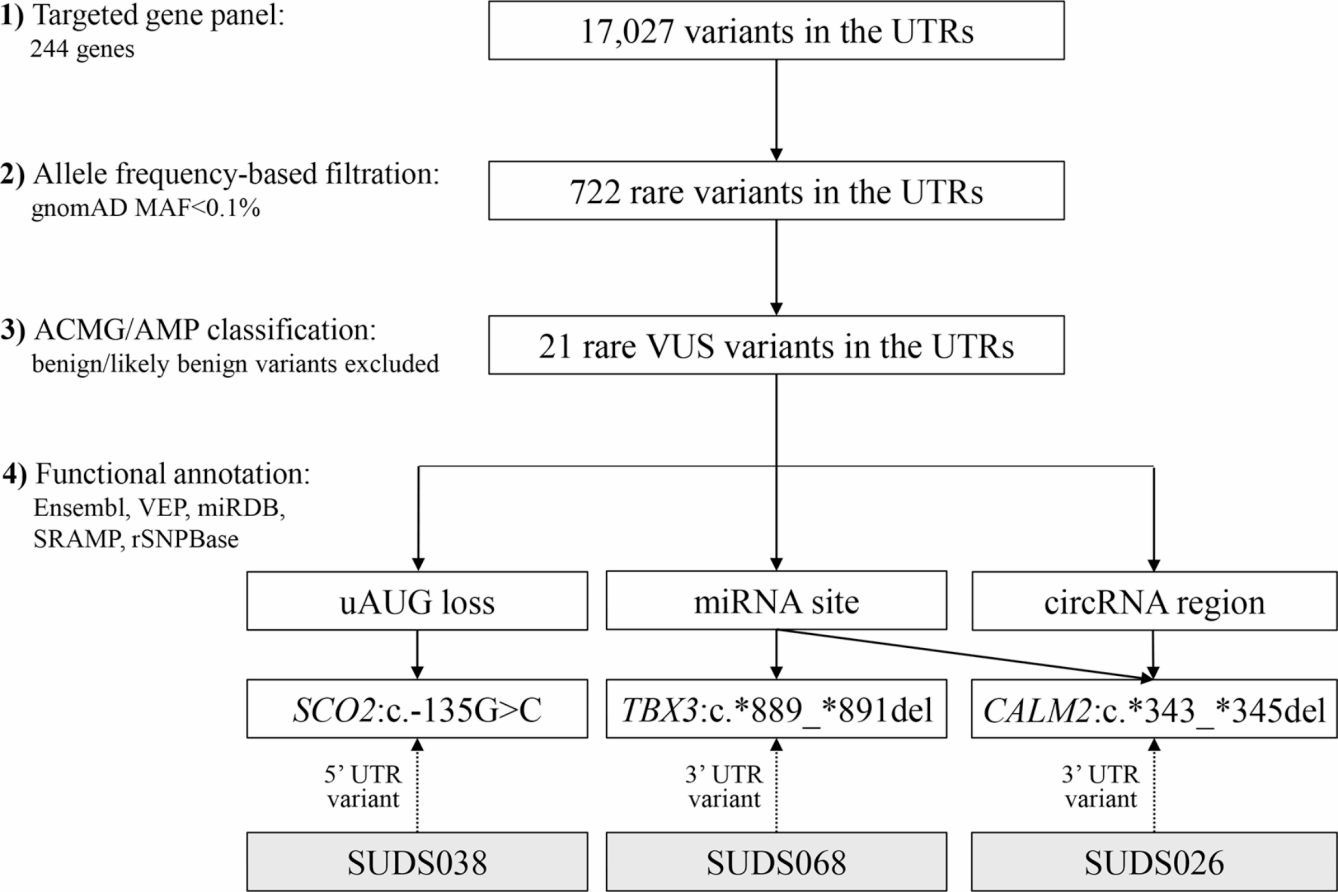
The filtering strategy of UTR variants and the number of remaining candidate variants at each step. MAF, minor allele frequency; ACMG/AMP classification based on Varsome v.11.6

Among the 17,027 variants identified in the UTRs of 244 genes investigated, there were only 21 rare variants (MAF < 0.1%) that met our requirements of ACMG/AMP classification. Through functional annotation, three variants were further found to overlap with potential regulatory elements (Fig. 1). As shown in Table 1, the three heterozygous variants were all classified as VUS with a conservation score ranging from 1.378 to 7.385. The variants were located in the 5’ UTR of one gene (*SCO2*) and in the 3’ UTR of another two genes (*CALM2* and *TBX3*). One of the variants identified in SUDS038 (*SCO2*, (NM_001169109.1):c.-135G > C) was extremely rare and has previously not been reported in the gnomAD database. The other two variants identified in SUDS026 (*CALM2*, (NM_001743.6):c.*343_*345del) and SUDS068 (*TBX3*, (NM_005996.4):c.*889_*891del) were also found to be ultra-rare (MAF < 0.01%).

**Table 1:** Variants with likely regulatory effect identified in the SUD cases

Case ID	HGSV ID	GRCh37 position	rsID	MAF a	Location	Coverage	HAF b	ACMG/AMP classification c		CoS d	ClinVar interpretation	Functional prediction			
								Verdict	Criteria			VEP	miRDB	SRAMP	rSNPBase
SUDS038	*SCO2*(NM_001169109.1):c.-135G> C	Chr22:50964796	-	-	5’UTR	42	0.45	VUS	PM2 PP3	1.378	-	uAUG loss	-	-	-
SUDS026	*CALM2*(NM_001743.6):c.*343_*345del	Chr2:47387570	rs1025341795	3.2E-05	3’UTR	32	0.47	VUS	PM2	7.385	-	-	miRNA site	-	circRNA region
SUDS068	*TBX3*(NM_005996.4):c.*889_*891del	Chr12:115108755	rs1215551312	3.2E-05	3’UTR	38	0.47	VUS	PM2	4.095	-	-	miRNA site	-	-

a MAF, minor allele frequency based on gnomAD Exomes v2.1.1

b HAF, heterozygous allele frequency

c ACMG/AMP classification based on Varsome v11.6

d CoS, conservation score based on PhyloP100way ver13. The site is predicted to be more conserved when the score is higher

**Fig. 2:**
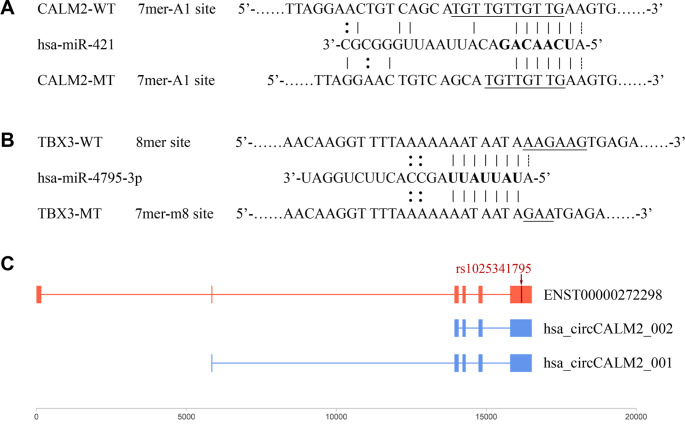
Predicted interaction between miRNAs/circRNAs and 3’ UTRs. The underlined nucleobases indicate the WT and MT alleles of the candidate variants, while the bold nucleobases indicate the seed regions of the miRNAs. **(A)** The *CALM2* variant could disrupt the binding of hsa-miR-421 to the 3’ UTR of this gene by directly interfering with base pairing. **(B)** The *TBX3* variant could alter the first nucleobase of the binding site and thus lead to a change of type (from 8mer site to 7mer-m8 site), which contributes to a decrease in specificity of the miRNA-UTR interaction. **(C)** The *CALM2* variant was found to overlap with the formation regions of two circRNAs. Red and blue color indicates the representative transcript of *CALM2* and known human circRNA regions, respectively

Although no records could be found for these variants in the NCBI ClinVar database, functional prediction revealed that the *SCO2* variant could lead to an uAUG loss, while the two 3’ UTR variants were both found to be located on predicted miRNA binding sites. As shown in Fig. 2A and Fig. 2B, hsa-miR-421 and hsa-miR-4795-3p were the miRNAs with the highest target scores for *CALM2* and *TBX3*, respectively, while the introduction of the candidate variants could either disrupt base pairing or lead to a change in binding site type. In addition, the *CALM2* variant was found to overlap with the formation regions of two circRNAs (Fig. 2C and Supplementary Table S3).

Among two of the three abovementioned SUD cases, several variants with likely functional effects in the coding regions of these 244 genes were previously identified [18]. Specifically, a likely pathogenic variant in exon 3 of *ACADS* (*ACADS*, (NM_000017.4):c.320G > A) and a VUS in exon 38 of *CACNA1C* (*CACNA1C*, (NM_199460.3):c.4604 C > G) was found in SUDS038, while another VUS in exon 326 of *TTN* (*TTN*, (NM_001267550.2):c.70349 A > T) was found in SUDS068. However, in SUDS026 no pathogenic variant or VUS was found in the coding region.

### The impact of candidate variants on gene transcriptional activity

**Fig. 3:**
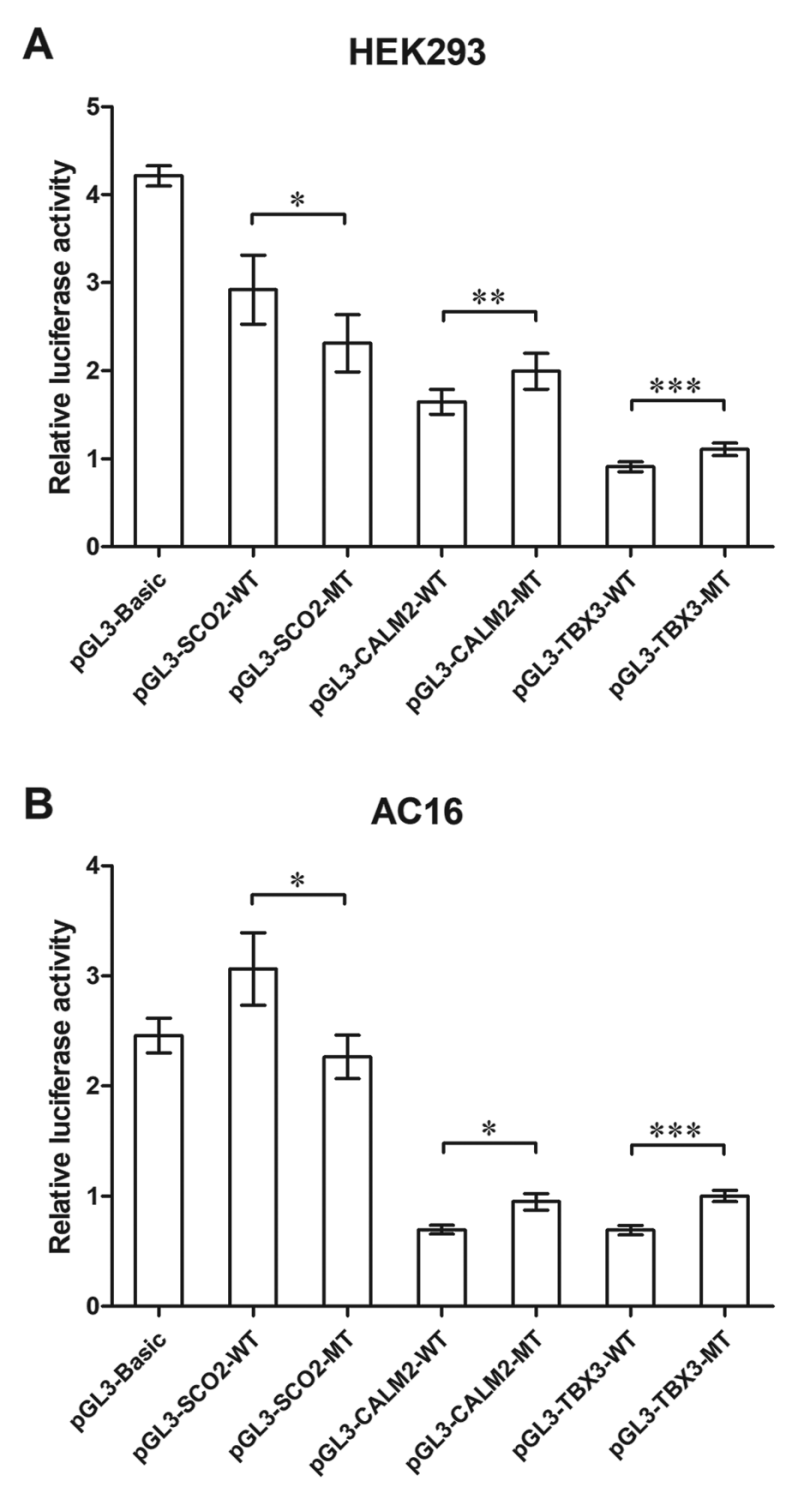
The impact of candidate variants on gene transcription measured by activity dual-luciferase reporter assay on HEK293 (A) and AC16 (B) cell lines. Significant difference was observed between the relative luciferase activity of cells transfected with pGL3-*SCO2*-WT and pGL3-*SCO2*-MT, cells transfected with pGL3-*CALM2*-WT and pGL3-*CALM2*-MT, as well as cells transfected with pGL3-*TBX3*-WT and pGL3-*TBX3*-MT (*N* = 9 per group; * *p* < 0.05; ** *p* < 0.01; *** *p* < 0.001)

In general, similar trends were observed in the two different cell lines (HEK293 and AC16) regarding the impact of candidate variants on gene transcriptional activity. As shown in Fig. 3, the relative luciferase activity of cells transfected with pGL3-*SCO2*-MT (HEK293: 2.31 ± 0.32; AC16: 2.26 ± 0.20) was significantly lower compared to that of cells transfected with pGL3-*SCO2*-WT (HEK293: 2.92 ± 0.39; AC16: 3.06 ± 0.33), suggesting that the candidate variant in the 5’ UTR of *SCO2* induced a reduction in its downstream gene expression. In addition, cells transfected with pGL3-*CALM2*-MT (HEK293: 1.99 ± 0.20; AC16: 0.95 ± 0.07) or pGL3-*TBX3*-MT (HEK293: 1.11 ± 0.07; AC16: 1.00 ± 0.05) showed a significant increase in terms of the relative luciferase activity compared to cells transfected with pGL3-*CALM2*-WT (HEK293: 1.65 ± 0.14; AC16: 0.70 ± 0.04) or pGL3-*TBX3*-WT (HEK293: 0.91 ± 0.05; AC16: 0.69 ± 0.04), indicating that the candidate variants in the 3’ UTR of *CALM2* and *TBX3* could disrupt certain regulatory elements of their upstream genes.

### Intolerance of genes to dosage effect

Based on the constraint metrics (Table 2), only pLI values for *CALM2* (0.921) and *TBX3* (0.989) were greater than 0.9. Furthermore, the LOEUF values for these two genes were close to or less than 0.35, indicating that *CALM2* and *TBX3* are likely to be intolerant to LOF variation, while *SCO2* is not. Regarding the intolerance of regulatory sequence variation, *CALM2* is in the upper 25th percentile of ncRVIS, suggesting that this gene is more intolerant to variation in the regulatory sequence compared to most other genes. Besides, *CALM2* and *TBX3* are both in the upper 25th percentile of ncGERP, indicating that these two genes have relatively strong dosage sensitivities due to their highly conserved non-coding sequences compared to the rest of the genome. In addition, the dosage sensitivity scores further verified that *TBX3* is very likely to exhibit haploinsufficiency. However, since no available data for *SCO2* and *CALM2* could be obtained from the ClinGen Genome Dosage Map, we were not able to judge whether these two genes are subject to triplosensitivity.

**Table 2:** Constraint metrics, intolerance indexes, and dosage sensitivity scores of the genes affected by candidate variants

Gene	Transcript	Constraint metrics		Intolerance of regulatory sequence variation				Dosage sensitivity scores		
		pLI	LOEUF	ncRVIS	ncRVIS percentile	ncGERP	ncGERP percentile	TS score	HI score	%HI
*SCO2*	ENST00000543927.1	0.000	1.764	-	-	-1.616	99.045	-	-	-
*CALM2*	ENST00000272298.7	0.921	0.368	-0.766	14.226	3.789	0.346	-	-	-
*TBX3*	ENST00000257566.3	0.989	0.281	0.670	84.238	0.914	21.449	0 (No Evidence)	3 (Sufficient Evidence)	3.050

pLI, gnomAD probability of being loss-of-function intolerant score. Values greater than or equal to 0.9 indicate that a gene appears to be intolerant of loss of function variation

LOEUF, gnomAD loss-of-function observed/expected upper bound fraction. Values less than 0.35 indicate that a gene appears to be intolerant of loss of function variation

ncRVIS, noncoding residual variation intolerance score. Genes with lower scores are more intolerant to variation in the regulatory sequence

ncGERP, noncoding genomic evolutionary rate profiling. Genes with highly conserved noncoding sequence relative to the rest of the genome are strongly correlated with gene dosage sensitivity

TS, Triplosensitivity score; HI, haploinsufficiency score; %HI, DECIPHER haploinsufficiency index. Values less than 10% predict that a gene is more likely to exhibit haploinsufficiency

## Discussion

It is well known that UTRs have comparable genomic footprints similar to coding regions and important regulatory roles in human diseases [10]. So far, a variety of different regulatory elements are known to be distributed in the UTR or upstream/downstream regions of gene coding sequences, such as uAUG sites [33], miRNA binding sites [34], and methylated CpG sites [35]. Regarding the identification of such regulatory elements, some could be predicted with specific algorithms based on characteristic sequences (e.g. the AUG initiation codon sequences or sequences matching to known miRNA seed regions), while some still lack a recognizable pattern and are difficult to identify by in-silico prediction. Therefore, functional assays remain an important way to confirm the potential effects of a given genetic variant on gene regulation. Since UTR variants usually cause disease through gene dosage effects, when looking for pathogenic UTR variants, it is important to focus on sequences that fall into regulatory elements that have well-established or functionally validated links to target genes. Besides, there should be well-documented associations between the target genes and phenotypes of interest. For SUD, the phenotypes could be short/prolonged QT intervals, ventricular fibrillation, conduction block, etc.

Currently, functional assays widely used to test the impact of non-coding variants on gene regulation include RNA sequencing, luciferase reporter assays, and chromosome conformation capture (3C) approaches. However, each assay has its own advantages and disadvantages. For example, RNA sequencing is the most direct way to detect aberrant gene expression, but fresh human tissue from exactly the same anatomical site of different individuals is not always available, not to mention that most forensic samples are highly degraded. Previous studies investigating human post-mortem gene expression have shown that mRNA degradation occurs in a tissue-specific manner and is associated with gene-specific properties [36, 37]. Salzmann et al. also demonstrated in their study on body fluids that some gene transcripts are more prone to degradation than others after deposition [38]. The stability discrepancies among different transcripts in degraded samples will make the comparison of RNA sequencing results even more difficult. In contrast, the luciferase reporter assay can avoid the influence of external factors, such as postmortem interval and individual variation. Nevertheless, the disadvantage of this method is that the assay system is artificial, which might not be able to accurately simulate in-vivo conditions. Regarding cell lines for the luciferase reporter assays, HEK293 and AC16 were used in parallel to increase the validity of our results. Due to the fact that the expression pattern of some regulatory RNAs are tissue specific [39], a representative cell line that shares the common expression regulatory mechanism with the studied tissue/organ is recommended. AC16 is a cell line derived from adult human ventricular cardiomyocytes and thus usually used to study developmental regulation of cardiomyocytes. In addition, HEK293 is a cell line derived from human embryonic kidney cells and currently widely used for functional studies due to its well-performance regarding reproducibility and transfection amenability. Previous studies have demonstrated that the combination of these two cell lines could improve the reliability of functional studies focusing on regulatory elements [40].

Among our candidate variants, only the one in the 5’ UTR of *SCO2* induced a reduction in gene expression, which is similar to the effect of LOF variants in the coding region of this gene. Previous studies have shown that uAUG-generating variants are under strong negative selection [33], suggesting a correlation between such variants and aberrant gene expression. As the encoding gene for a metallochaperone involved in the biogenesis of cytochrome *c* oxidase (COX) subunit, *SCO2* dysfunction could cause COX deficiency, resulting in reduced mitochondrial oxidative ATP production capacity [41]. Previous studies have proposed that mutations in *SCO2* are associated with infantile encephalocardiomyopathy [42] and hypertrophic cardiomyopathy [43]. However, a comprehensive assessment of this gene’s constraint metrics, intolerance indexes, and dosage sensitivity scores indicated that the aberrant expression of *SCO2* will not likely cause fatal disease alone. Thus, we assume that the *SCO2* variant identified in this study is at most a contributing factor rather than the main cause of SUD, even though it could lead to a moderate expression change in *SCO2*. SUDS038 was previously found to also carry a likely pathogenic variant in the coding region of *ACADS* and a VUS in the coding region of *CACNA1C* [18]. Therefore, it is possible that the coding variants and UTR variants have all contributed to the death of this individual to varying degrees, as suggested by the multifactorial model of cardiac genetic diseases [44].

The 3’ UTR variants identified in *CALM2* and *TBX3* could disrupt their binding to certain miRNAs, thereby up-regulating gene expression, leading to a similar effect as GOF variants in coding regions. However, the constraint metrics could only provide evidence that *CALM2* and *TBX3* are intolerant to LOF variation. Subsequent ncRVIS and ncGERP analysis revealed that *CALM2* and *TBX3* are intolerant to variation in their regulatory sequences and might have strong dosage sensitivities. The dosage sensitivity scores further confirmed that *TBX3* is subject to haploinsufficiency, while it remains questionable whether these two genes also exhibit triplosensitivity. Taken together, these data highlight the strong deleterious effect of aberrant expression of *CALM2* and *TBX3*. As a calmodulin encoding gene, *CALM2* is associated with severe early-onset LQTS, which can cause life-threatening ventricular arrhythmias [45]. According to a summary of published pathogenic missense variants in this gene [46], both variants that increase and decrease its affinity for Ca^2+^ have been identified in LQTS cohorts, suggesting that either LOF or GOF variants in *CALM2* are phenotype-related. Therefore, variants that lead to an overexpression of this gene, as shown in this study, are also very likely to be pathogenic. More importantly, our previous study focusing on the coding region did not identify any pathogenic variant or VUS in SUDS026, further strengthening the significance of this single positive finding. However, it remains unclear whether this UTR variant can be considered the sole cause of death until we better understand to what extent the decrease of *CALM2* is severe enough to have fatal consequences. The *TBX3* gene is a well-known member of the ancient T-box gene family, which is conserved across a wide range of species. It is a critical developmental regulator of several structures, including the heart [47]. Moreover, Frank et al. have shown that *TBX3* is required for functional maturation and post-natal homoeostasis of the conduction system in a highly dosage-sensitive manner [48]. Taken together with our findings, it is reasonable to assume that in this case (SUDS068), a joint effect of this variant and another previously identified VUS in the *TTN* coding region could be responsible for the lethal arrhythmia.

It is noteworthy that the ACMG/AMP guidelines were applied for the initial variant screening of the massive UTR variants identified in our SUD cohort, even though many of these existing rules relate specifically to coding region variants. As proposed by Ellingford et al. [49], some of the rules from Richard et al. [24] can be applied directly to variants in non-coding regions, without the need for additional considerations. These include the use of frequency information (BA1, BS1, BS2, and PM2), up-weighting of confirmed *de novo* variants (PM6 and PS2), and incorporation of co-segregation evidence (PP1 and BS4). But some other rules from Richard et al. require modifications in terms of strength of evidence and manual curation of triggering conditions (Table 3). In our study, PM2 (absent from controls or at extremely low frequency) was met for the three candidate variants due to their low frequencies in the general population. For computational prediction, PP3 (multiple lines of computational evidence support a deleterious effect on the gene or gene product) was met for *SCO2* (NM_001169109.1):c.-135G > C based on in-silico prediction (MetaRNN score = 0.82, which is within the range of pathogenic supportive evidence), while the other two variants were not assigned with a high deleterious score by the prediction tools integrated in Varsome. A possible explanation could be that many pathogenic mutations that act via dominant-negative or GOF mechanisms are likely to be missed by current variant prioritization strategies [50]. In addition to computational prediction, functional evidence is also important in assessing whether a non-coding variant is pathogenic or benign. As our results from the dual-luciferase reporter assay suggest that the three variants all have significant effects on gene expression, manual curation to activate PS3 (well-established in-vitro or in-vivo functional studies supportive of a damaging effect on the gene or gene product) should be acceptable.

**Table 3:** Applicability of ACMG/AMP rules for non-coding region variants [49]

	Pathogenic		Benign	
Rules applicable for non-coding region variants directly	PS2	*De novo* variants (maternity and paternity confirmed)	BA1	Allele frequency > 5%
	PS4	Prevalence significantly increased in cases over controls	BS1	Allele frequency too high for disorder
	PM2	Absent/low-frequency in population databases	BS2	Observed in healthy indivduals with full penetrance expected
	PM4	Lead to protein length change	BS4	Non-segregation with disease
	PM6	*De novo* variants (assumed)	BP2	Observed in *trans* with a dominate variant, or in *cis* with a pathogenic variant
	PP1	Co-segregation with disease	BP5	Found in case with alternative molecular basis
	PP4	Patient’s phenotype or FH highly specific for gene		
Rules applicable for non-coding region variants with modification	PVS1	Null variant in a gene where LOF is a known mechanism for disease	BS3	Well established functional studies show no deleterious effect
	PS1	Splicing variant at same nucleotide as established pathogenic variant	BP4	Computational evidence suggests no impact
	PS3	Well-established functional studies show deleterious effects		
	PM1	Mutational hotspot or well-established functional domain		
	PM3	In *trans* with a pathogenic variant for recessive disorders		
	PM5	Same predicted impact as established pathogenic variants		
	PP3	Computational evidence supports deleterious effect		
Rules not applicable for non-coding region variants	PP2	Missense in gene with low rate of benign missense variants	BP1	Missense in genes where only truncating cause disease
	PP5	Reputable source reports as pathogenic	BP3	In-frame indels in repetative region without known function
			BP6	Reputable source reports as benign
			BP7	Synonymous variant with no predicted splicing impact

There are also some limitations in our study. First, not all currently available in-silico prediction tools were applied for the functional annotation of the UTR variants, since some are open source scripts that can only run on a specific programming language or environment. Therefore, some regulatory variants with functional effects may have been missed in this study. However, as new prediction algorithms continue to emerge rapidly through iterative improvements, the in-silico tools we used are also likely to be outdated quickly. With this in mind, periodic re-analysis of molecular autopsy results is recommended, as there may be significant updates in variant interpretation in the future [51, 52]. Second, although the candidate variants seem to cause aberrant gene expression, we were unable to confirm their actual contribution to SUD due to the lack of conclusive evidence in terms of gene dosage effect. In future studies, more explicit associations between up/down-regulation of previously proposed SUD susceptibility genes and specific phenotypes deserve to be better clarified to identify the dosage-sensitive genes that are intolerant to variation in their regulatory sequences.

In conclusion, by functional analysis of the UTR variants from the molecular autopsy findings in our SUD cohort, we identified three variants with high confidence of pathogenicity in the UTRs of *SCO2*, *CALM2* and *TBX3*. Our strategies for UTR variant prioritization and functional annotation could be considered as a practical way of interpreting molecular autopsy findings in the non-coding sequences, which will improve the understanding of the pathogenicity of a significant proportion of VUS identified in SUD cohorts.

## Electronic supplementary material

Below is the link to the electronic supplementary material.

Supplementary Material 1

## Data Availability

All data will be provided upon request to the first or corresponding authors.
